# Measuring Claw Conformation in Cattle: Assessing the Agreement between Manual and Digital Measurement

**DOI:** 10.3390/ani5030379

**Published:** 2015-08-06

**Authors:** Linda J. Laven, Libin Wang, Corey Regnerus, Richard A. Laven

**Affiliations:** 1 IVABS, Massey University, Palmerston North 5321, New Zealand; E-Mails: l.j.laven@massey.ac.nz (L.J.L.); regnerusc@gmail.com (C.R.); 2 Gansu Agricultural University, Lanzhou 730070, China; E-Mail: wanglb@gsau.edu.cn

**Keywords:** claw conformation, limits-of-agreement, method comparison, digital image, manual measurement

## Abstract

**Simple Summary:**

Claw conformation is commonly measured in cattle. It can be measured at cow-side or, by using digital images, on a computer. This study compared, for five conformational features of the claw, measurements made directly from the hoof with those made from a digital image of the same claw. Of the five measures, only one, toe angle, had results where agreement was good enough for the two measurements to be used interchangeably. The variation in differences between the digital and manual results for the other four measures was too great for them to be used interchangeably. When measuring claw conformation, more attention needs to be paid to the method used and it should not just be assumed that a different technique would have produced the same result.

**Abstract:**

Five measurements of claw conformation (toe angle, claw height, claw width, toe length and abaxial groove length) taken directly from the hoof were compared with the measurements taken from digital images of the same claws. Concordance correlation coefficients and limits-of-agreement analysis showed that, for four of the five measures (claw height, claw width, toe length and abaxial groove length), agreement was too poor for digital and manual measures to be used interchangeably. For all four of these measures, Liao’s modified concordance correlation coefficient (mCCC) was ≤0.4, indicating poor concordance despite Pearson’s correlation being >0.6 in all cases. The worst concordance was seen for toe length (mCCC = 0.13). Limits-of-agreement analysis showed that, for all four measures, there was a large variation in the difference between the manual and digital methods, even when the effect of mean on difference was accounted for, with the 95% limits-of-agreement for the four measures being further away from the mean difference than 10% of the mean in all four cases. The only one of the five measures with an acceptable concordance between digital and manual measurement was toe angle (mCCC = 0.81). Nevertheless, the limits-of-agreement analysis showed that there was a systematic bias with, on average, the manual measure of toe angle, being 2.1° smaller than the digital. The 95% limits-of-agreement for toe angle were ±3.4°, probably at the upper limit of what is acceptable. However, the lack of data on the variability of individual measurements of claw conformation means that it is unclear how this variability compares to measurement of toe angle in the same animal using the same or a different manual technique.

## 1. Introduction

It has long been recognized that being able to accurately assess conformational traits could be of significant benefit when determining selection policies and gauging the impact of factors, such as the environment and nutrition, on the hoof and on the risk of lameness [1]. Claw horn quality has been defined as the “*product of horn characteristics*, *claw shape and the anatomy and physiology of inner structure*” [2]. There is, thus, a need to employ accurate and repeatable measurement methods for trait evaluation in order to avoid the inaccuracies and biases inherent in systems based simply on visual appraisal [3]. 

**Figure 1:**
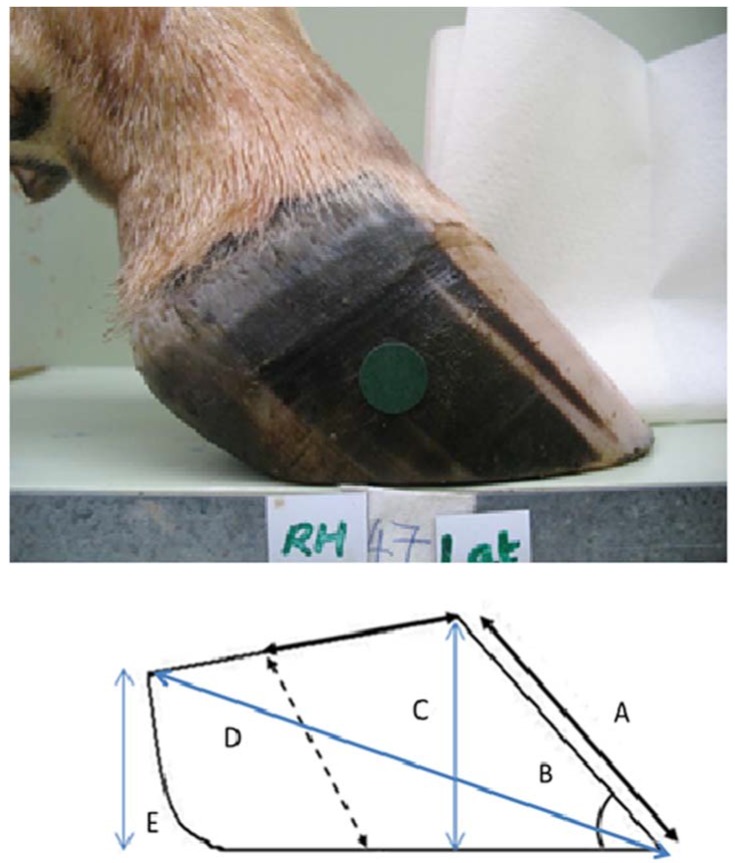
Photograph of right hind, lateral claw from the abaxial aspect, and a schematic illustration of five conformational features: (**A**) length of dorsal border; (**B**) toe angle; (**C**) toe height; (**D**) diagonal claw length and (**E**) heel height, dashed line represents abaxial groove.

**Figure 2:**
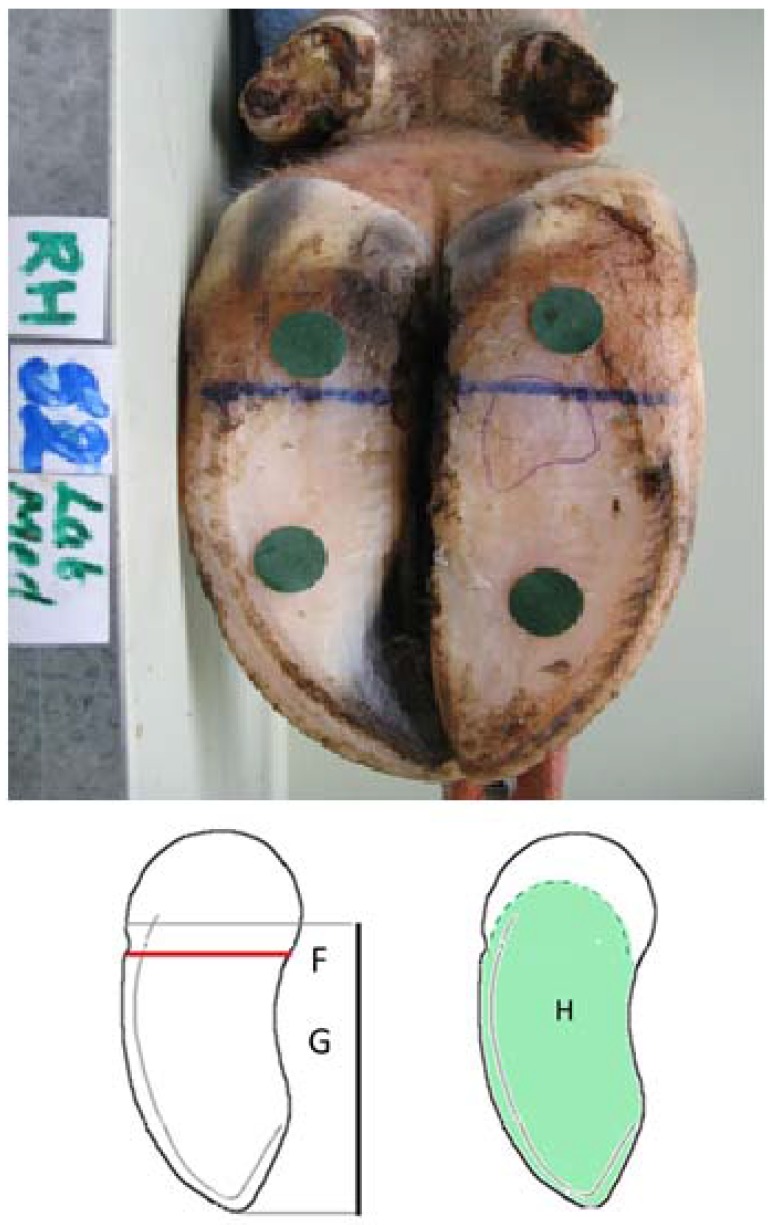
Photograph of the right hind foot from the plantar aspect, and a schematic illustration of three conformational features: (**F**) claw width; (**G**) claw length and (**H**) surface area in contact with the ground.

In a review [3] eight traits were identified as being commonly used in the classification of bovine claws [3]: toe angle, length of dorsal border, heel height, diagonal length, claw length, toe height, claw width and surface area of the sole in ground contact. Figure 1 illustrates the traits that can be seen when viewing the abaxial aspect of the claw, while Figure 2 illustrates those that can be seen from the palmar/plantar aspect.

A number of methods have been used to measure conformational traits. For example, toe angle has been measured in the weight-bearing limb [4] or in the lifted foot in a foot crush [5], or a tilt table [6], with measurements made using a goniometer [7], a customized commercial protractor [4], a carpenter’s profile gauge [8], and an engineer’s electronic angle finder [5]. Additionally, measurements of toe angle have been made from digital images rather than directly from the foot [9,10].

Different means or modes of data collection could affect the results of measurements of conformational traits; nevertheless, some studies of hoof conformation have merely reported that a measurement (e.g., toe angle) was made, and have not reported a detailed method (e.g., [11,12]). More attention to the method used to measure conformational traits is required; in particular, direct comparisons of methods are essential in order to establish whether the methods are interchangeable and whether conclusions based on one technique would also apply if another technique had been used.

One area of particular concern is the comparison between measurements from digital images with those taken directly from the hoof. The aim of this study was to compare the results of measurements made directly from the hoof post mortem with the results from measurements made from digital images of the same hooves.

## 2. Materials and Methods

The distal limbs were collected from 54 dairy cows. Each limb was identified on removal from the carcass using an elastic band of a specified color (red: right front, yellow: left front, blue: left hind and green: right hind), which remained with the limb throughout processing. The limbs were then washed to remove any mud or manure, and a latex glove was placed over the sawn end of the limb to prevent moisture loss. All four limbs from each animal were placed in individual plastic bags, sealed with a cable tie, and stored at 3 °C to 5 °C until anatomical measurements were completed

The cows came from three sources: (1) a local abattoir (18/54); (2) the Massey University post mortem room (animals with a cause of death unrelated to lameness) (23/54); (3) non-lame cull dairy cows euthanized on farms for reasons unrelated to this study (13/54).

Five conformational variables were recorded

**Figure 3:**
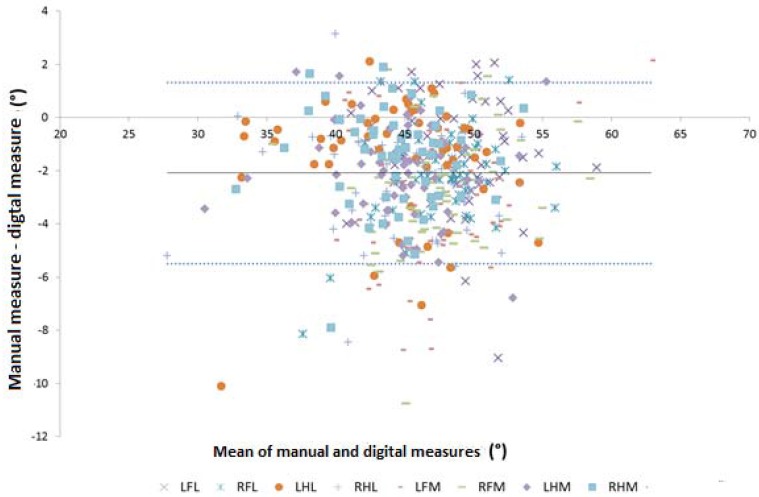
Mean *vs.* difference plot for toe angle as measured manually and digitally. LFL, left front lateral claw; RFL, right front lateral, LHL, left hind lateral; RHL, right hind lateral; LFM, left front medial; RFM, right front medial; LHM, left hind medial; RHM, right hind medial (Interpretation: Solid line is the mean difference (2.1°); dashed lines are the upper and lower 95% limits-of-agreement (1.3 and −5.5°, respectively)).

**Figure 4:**
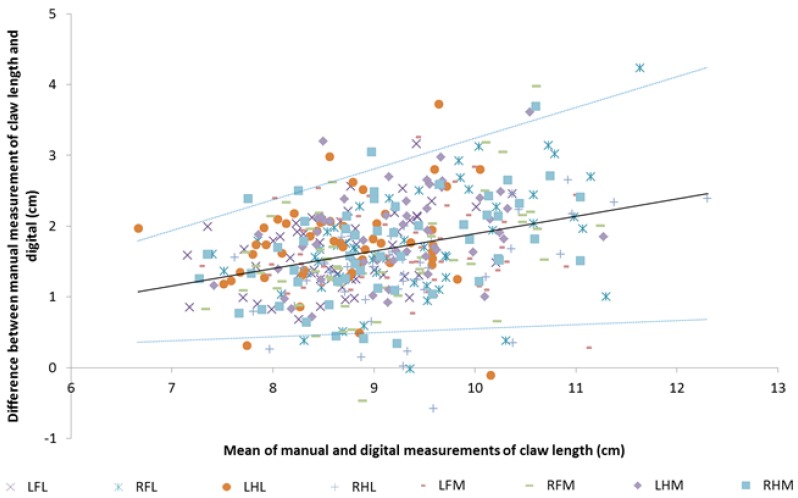
Mean *vs.* difference plot for claw length as measured manually and digitally. LFL, left front lateral claw; RFL, right front lateral, LHL, left hind lateral; RHL, right hind lateral; LFM, left front medial; RFM, right front medial; LHM, left hind medial; RHM, right hind medial (Interpretation: Solid line is the expected difference from the mean; dashed lines are the upper and lower 95% limits-of-agreement for that line of best fit).

(1) Length of dorsal border from skin: horn junction at coronary band to apex of toe (Measure A; Figure 1);
(2) Toe angle: Angle of dorsal border to weight-bearing surface; (Measure B; Figure 1);
(3) Length of the abaxial groove from the coronary band to base of claw (see Figure 1);
(4) Claw width on the palmar/plantar surface, at widest point of claw in area of sole/bulb junction. (Measure F; Figure 2);
(5) Claw length on the palmar/plantar surface from apex of toe to point where ground contact is lost (Measure G; Figure 2).

The first measurements made were made directly from the hoof. All such measurements, except for toe angle, were made using a flexible tape measure. Toe angle was measured using an engineer’s angle finder. All measurements were repeated twice and the mean value used for modeling. For linear measures, the difference between the two estimates was never more than two millimeters, whilst toe angle replicates were within one degree of each other. All measurements were made by the same observer (LJL).

Digital photographs were then taken with each limb in a standardized position within a bespoke photographic box, which allowed the foot to be carefully positioned and clamped at a constant distance from the mounted camera (Power Shot A640, Canon New Zealand, Auckland, New Zealand). Adhesive calibration dots of 1.4 cm in diameter were placed on each claw; one in the toe region, one in the heel region, and one on the lateral wall [13,14] (see Figure 1 and Figure 2). The images taken were an abaxial projection of each claw and the sole surface of both claws. Images were taken on the macro setting, with the hoof image filling the display.

The same measurements that had been made from the hoof were then made from the digital images using the same instructions as had been used on the hoof. The data were measured using ImageJ (NIH, MD, USA) with the landmarks being identified and then, for the linear measures, the distance between them being measured from landmark at the top of the image to the one at the bottom and then the reverse. All measurements were made by the same observer (WL).

### Statistical Analysis

The data were analyzed in two ways. Firstly, for each measure, the data for all claws taken directly from the foot (“manual”) were compared to the data from all claws measured from a digital image (“digital”) using Liao’s modified version of Lin’s concordance correlation coefficient (mCCC) [15]. This uses the mean and variance of the two measurements to produce a numerical value that reflects how close the fitted regression line for the two methods is to the line of identity. Liao’s modification uses two points rather than one to examine the area between the two lines, fixing the slope of the regression line in relation to the line of identity [15]. The equation used was:

mCCC = p * 4σ_1_σ_2_ − *p*(σ_1_^2^ + σ_2_^2^)/(2 − *p*)( σ_1_^2^ + σ_2_^2^) + (μ_2_ − μ_1_)^2^

where σ is the variance, μ the mean, and *p* the Pearson’s correlation coefficient.

Limits-of-agreement plotting was then used to evaluate, for each measure, the agreement between the results from measures taken directly from the foot with those taken from a digital image [16]. This analysis was undertaken with all the data from all the claws amalgamated together, accounting for repeated measures per animal [17]. For data where mean and difference were significantly related, limits-of-agreement were calculated as shown in [18].

A linear mixed model with the difference between the manual and digital measurement of each claw as the dependent variable, with claw (e.g., right hind lateral) as a repeated measure and claw and mean of the manual and digital measurement of each claw (plus their interaction) as the independent variables, was then used to identify whether claw had a significant effect on the difference between the digital and manual measures. An unstructured covariance structure was used for this model. The goodness-of-fit of the mixed models were assessed by checking the residuals for normality and for influential outliers. 

To identify whether claw affected the variance of the difference between the manual and digital measurements the model was rerun with compound symmetry (which assumes constant variance across all groups) as the covariance structure. The difference between the −2 log likelihood (−2LL) results for each covariance structure was then calculated. This statistic was then tested for significance using the Chi-squared test, with the number of degrees of freedom being equal to the difference in number of parameters between each model. All analyses were undertaken using SPSS Statistics 21 (IBM, New York, NY, United States).

## 3. Results

The descriptive data for each of the measures using both methods and the Pearson’s correlation coefficient and the mCCC are shown in Table 1. Data were available from 429 claws (54 cows) for toe angle, 428 claws for claw and toe length (54 cows), 427 claws (54 cows) for abaxial groove length, and 366 claws (46 cows) for claw width. Note: limbs from eight cows were ineligible for lesion score recording and so the palmar/plantar views needed for digital width assessment were not captured for these claws. 

**Table 1:** Comparison of mean and standard deviation for five claw conformation measures measured directly from the hoof and from a digital image. Data were from claws from 54 dairy cows (except claw width where *n* = 46).

	From Hoof	From Digital Image	*p*	mCCC
	Mean (St Dev)	Mean (St Dev)		
Toe angle (°)	45.1 (4.68)	47.2 (4.54)	0.89	0.82
Toe length (cm)	7.96 (0.6)	7.15 (0.54)	0.61	0.13
Claw length (cm)	9.92 (1.05)	8.25 (0.8)	0.74	0.21
Claw width (cm)	4.56 (0.51)	4.98 (0.46)	0.78	0.27
Abaxial groove length (cm)	5.6 (0.7)	5.03 (0.74)	0.71	0.4

*p*, Pearson’s correlation coefficient; mCCC, Liao’s modification of Lin’s concordance correlation coefficient; St Dev, standard deviation.

### 3.1. Toe Angle: Limits of Agreement Analysis

The limits of agreement plot for toe angle for all claws is shown in Figure 3. On average the manual measure was 2.1° smaller than the digital measure. This difference was not significantly associated with the mean of the two measures (R^2^ = 0.0004). The limits-of-agreement were ±3.4° around this mean; *i.e.*, 95% of the differences between the two measures (manual—digital) were between 1.3° and 5.5°.

Claw was significant in the mixed model (*p =* 0.026), although mean of the manual and digital measure and the interaction between this mean and claw were not (*p =* 0.34 and 0.06, respectively). Mean difference was significantly different for the medial claws in the two front feet (−3.2° (95% confidence interval, CI, −3.9 to −2.6) and −2.9° (95% CI −2.5 to −2.3), for left and right foot, respectively) compared to the other six claws (mean difference for all six claws was −1.8° (95% CI −1.6 to −2.03)). There was no significant effect of claw on the variance of the difference between the two measures (−2LL difference 36.4; degrees of freedom (df.) 18; *p =* 0.36).

### 3.2. Toe Length: Limits of Agreement Analysis

On average, the manual measure was 0.8 cm longer than the digital measure; this difference did not vary significantly with the mean of the two measures (R^2^ = 0.006). The limits-of-agreement were ±0.96 cm around this mean; *i.e.*, 95% of the differences between the two measures (manual—digital) were between 1.76 cm and −0.16 cm.

Claw, mean of the manual and digital measurement and their interaction were all significant in the mixed model (*p* ≤ 0.002 for main effects, *p =* 0.022 for interaction). The estimated marginal mean difference for the right front medial claw (1.17 cm) was significantly greater than the mean difference of all the other claws (*p* ≤ 0.002, except for the comparison with the left hind lateral claw (*p =* 0.038)). The two claws with the smallest mean difference were the lateral claws of the right hind and right front feet (mean = 0.55 and 0.68 cm, respectively. These were significantly lower than the mean differences for the right front medial and left hind lateral claw (*p* < 0.001 and 0.036, respectively).

The regression of difference against mean was not significant for any of the claws except for the right front medial claw (R^2^ = 0.127). The regression equation for the right front medial claw was:

∆ = 0.481 (95%CI 0.148 – 0.814) * μ − 2.479 (95%CI −4.95 – −0.09)

where ∆ is the difference between the two measures, and μ is their mean.

The effect of claw on the variance of the difference between the two measures was significant at the 5% level (−2LL difference 51.8; df. 18; *P =* 0.026); the standard deviation of the difference between the two methods was greatest for the right front medial claw (0.56 cm) and lowest for the right hind medial claw (0.41 cm).

### 3.3. Claw Length: Limits of Agreement Analysis

On average the manual measure was 1.67 cm longer than the digital measure; this difference varied significantly with the mean of the two measures (R^2^ = 0.107). The association between mean and difference was:

∆ = 0.246 (95%CI 0.179 – 0.314) * μ − 0.564 (95%CI −1.18 – 0.054)

where ∆ is the difference between the two measures, and μ is their mean. Analysis of the residuals showed the standard deviation of the association was 0.0967* mean −0.274. These limits of agreement are shown in Figure 4. Based on this model, for a manual claw length measurement of 7 cm, the mean digital result would be 5.8 cm, with 95% of results being between 5.1 and 6.6 cm; for a manual measurement of 10 cm, the equivalent figures would be a mean of 8.1 cm, with 95% of results between 6.8 and 9.5 cm.

Claw, mean of the manual and digital measurement and their interaction were all significant in the mixed model (*p* < 0.018). The estimated marginal mean difference for the right hind lateral claw (1.37 cm) was the lowest mean difference and was significantly lower than that for all four claws from the left hand side (*p* ≤ 0.028). The regression of difference against mean was significant for all claws except for the left hind lateral and left front medial claws (*p =* 0.21 and 0.89, respectively). There was no significant difference between the regression equations for the remaining six claws (*p* > 0.01). The effect of claw on the variance of the difference between the two measures was almost significant at the 5% level (−2LL difference 48.2; df.18; *p =* 0.054); the standard deviation of the difference between the two methods was greatest for the left front lateral claw (0.78 cm) and lowest for the left hind lateral claw (0.53 cm).

### 3.4. Claw Width: Limits of Agreement Analysis

On average the manual measure was 0.41 cm narrower than the digital measure; this difference varied significantly with the mean of the two measures (R^2^ = 0.03). The association between mean and difference was:

∆ = 0.123 (95%CI 0.051 – 0.195) * μ − 1 (95%CI −1.35 – −0.659)

where ∆ is the difference between the two measures, and μ is their mean. Analysis of the residuals showed the absolute residual was not significantly associated with the mean of the two claw width measurements (R^2^ = 0.037), so the limits of agreement ran parallel to the line of best fit for the mean-difference plot with the same slope (0.123) but a constant of –1.61 for the lower limit and −0.39 for the upper limit. Based on this model, for a manual claw width measurement of 3.5 cm, the mean digital result would be 4.1 cm, with 95% of results being between 3.5 and 4.7 cm; for a manual measurement of 6 cm, the equivalent figures would be a mean of 6.3 cm, with 95% of results between 5.7 and 6.9 cm.

Claw, mean of the manual and digital measurement and their interaction were all non-significant in the mixed model (*p =* 0.88, 0.1, and 0.76, respectively). The effect of claw on the variance of the difference between the two measures was not significant at the 5% level (−2LL difference 36; df.18; *p =* 0.37).

### 3.5. Abaxial Groove Length: Limits of Agreement Analysis

On average the manual measure was 0.5 cm longer than the digital measure; this difference varied with the mean of the two measures (R^2^ = 0.014) but, in contrast to claw width and length and toe length the association was negative:

∆ = −0.096 (95%CI −0.175 – −0.018) * μ + 1.022 (95%CI 0.609 – 1.44)

where ∆ is the difference between the two measures, and μ is their mean. Analysis of the residuals showed the standard deviation of the association was 0.07 * mean + 0.16. Based on this model, for a manual abaxial groove length measurement of 4 cm, the mean digital result would be 4.6 cm, with 95% of results being between 3.8 and 5.5 cm; for a manual measurement of 7 cm, the equivalent figures would be a mean of 7.8 cm, with 95% of results between 6.4 cm and 9.25 cm.

**Table 2:** Results of the limits-of-agreement analyses for five claw conformation measures measured directly from the hoof and from a digital image. Data were from claws from 54 dairy cows (except claw width where *n* = 46).

	Mean Difference *	Difference Associated with Mean ^†^	Limits-of-Agreement *^,†^	Difference Associated with Claw ^‡^	Variance Associated with Claw ^‡^
Toe angle (°)	−2.1	No (R^2^ = 0.0004)	−5.5 to 1.3	Yes (*p* = 0.026)	No (*p* = 0.36)
Toe length (cm)	0.8	No (R^2^ = 0.006)	−0.16 to 1.76	Yes (*p* < 0.001)	Yes (*p* = 0.026)
Claw length (cm)	1.67	Yes (R^2^ = 0.127)	At 7 cm: 5.1 to 6.6 At 10 cm: 6.8 to 9.5	Yes (*p* = 0.001)	No (*p* = 0.054)
Claw width (cm)	−0.41	Yes (R^2^ = 0.03)	At 3.5 cm: 3.5 to 4.7 At 6 cm: 5.7 to 6.9	No (*p* =0.88)	No (*p* =0.37)
Abaxial groove length (cm)	0.5	Yes (R^2^ = 0.014)	At 3.5 cm: −0.2 to 1.5 At −0.6 to 2.25	Yes (*p* < 0.001)	Yes (*p* = 0.003)

* manual - digital; **^†^** from mean *vs.* difference plot; **^‡^** from mixed model analysis.

Claw, mean of the manual and digital measurement and their interaction were all significant in the mixed model (*p* < 0.020). The largest estimated marginal mean differences were recorded for the right front lateral claw and left front medial claws (0.82 cm and 0.73 cm, respectively). These differences were significantly larger than those recorded for both the claws on the right hind foot and the left hind lateral claw *p* ≤ 0.026). The regression of difference against mean was significant only for the lateral claws of the front feet (*p* ≤ 0.008). For both of these feet the association between mean and difference was strongly negative (slope of the regression line was −0.33 and −0.43, for left and right foot, respectively).

The effect of claw on the variance of the difference between the two measures was highly significant (−2LL difference 48.2; df.18; *p* < 0.0003). The standard deviation of the difference between the two methods was greatest for the right front lateral claw (0.7 cm) and lowest for the left hind medial claw (0.3 cm).

The results of these analyses are summarized in Table 2.

## 4. Discussion

These data clearly show that except for toe angle, measurements made on the same feet directly from the hoof (“manual”) were not interchangeable with measurements made using imageJ and digital photograph (“digital”), even though the photographs were standardized by using a bespoke photographic box, which ensure that the hoof was photographed in a consistent position at a consistent distance from the camera. 

Although Pearson’s correlation was relatively good (r > 0.7) for 4/5 measures, the mCCC showed that this association did not mean that the measures agree; except for toe angle which was had good concordance (mCCC = 0.81), all mCCC were ≤0.4 indicating very poor concordance. This was further emphasized by the limits-of agreement analysis. 

For toe angle, as there was no association between mean and difference, a simple limits-of-agreement could be created, with 95% of differences between manual and digital being between 1.3 and −5.5°. Thus if the bias identified by the plot (−2.1°; see Figure 3) is included; 95% of differences would be between ±3.4°, with 47% of results between ±1°. Although not ideal, it is unlikely that such differences would have a major impact on conclusions made with digital rather than manual data, particularly as toe angle is one of the most difficult measures to accurately measure in the live cow.

For toe length, there was also no association between mean and difference which meant simple limits of agreement could be calculated; *i.e.*, between 1.76 and −0.16 cm or ± 0.96 if the 0.8 cm bias was included. This is relatively much larger than the difference seen in toe angle and strongly indicates that using digital images rather than manual measurement could significantly alter conclusions as to whether toe length was short or long. It is likely that this large difference was principally because it was much more difficult to establish the landmarks needed for this measurement on a digital image, particularly the horn/skin junction, compared to doing so on a hoof; however curvature of the claw may also have resulted in some of the differences.

For the other three measures, mean and difference were related. This meant that there was no simple way to calculate the bias as the difference changed dependent on the size of the measure. However limits-of-agreement could still be calculated, allowing an assessment of the level of agreement between the two measures. For claw width the limits-of-agreement were consistent across the range of measures seen in this study. This meant that the limits of agreement were ±0.6 cm around the regression line. As this was more than 10% of the value of claw width in these cows, this meant that digital and manual measures were not interchangeable. Again, ability to identify landmarks may have had an impact on the size of this difference, but as the landmarks for this measure are indistinct in the foot, repeatability may be an issue for this measure even when measured directly from the cow.

For claw length, limits-of-agreement increased as claw length increased so were ±0.75 cm at 7 cm but ±1.4 cm at 10 cm. The abaxial limits-of-agreement also increased as size increased from ±0.9 cm when mean claw length was 4 cm to ±1.4 cm when it was 7 cm. Thus, for both of these measures, even if the association between mean and difference could be eliminated by transformation these limits-of-agreement are just too large for the two measures to be interchanged.

Analysis at the claw level showed that the agreement between measures varied significantly by claw. For example for toe length marginal mean difference for the right front medial claw was almost twice that of the right hind lateral claw (1.17 cm *vs.* 0.55 cm, respectively). Furthermore, claw affected variance; for example for abaxial groove length the standard deviation of the difference between the two methods for the right front lateral claw (0.7 cm) was double that for the left hind medial claw (0.3 cm), equivalent to a doubling of the limits of agreement. The analysis by claw clearly shows that simply comparing means across all claws is not sufficient to establish agreement, even if the repeated measures nature of the data is taken into account. Further investigation is required to identify the reason for these differences between claws.

This analysis has shown that digital and manual measurements are not necessarily interchangeable, even if the bias can be calculated, as agreement was poor for four out of five measures. One key issue with this analysis is that the landmarks used in the manual measurements were not necessarily the same as those used in the digital measures; marking the landmarks on the hoof prior to photographing could therefore improve the agreement. This suggestion needs confirming.

Another issue highlighted by this study is the general lack of data on the size of the variability in claw measurements taken by a single person using the same method on multiple occasions or that between measurements by multiple people measuring the same claw using the same method. Several studies have reported repeatability based on intraclass correlation coefficients (ICC) for either multiple observers using the same methods or the same observer measuring the same cow multiple times (e.g., [1,19,20,21]). ICC are a ratio of the variability between subjects to the total variability [22], so they do not provide information on the magnitude of the variation between raters or measurements (*i.e.*, how big the differences are on average and how variable they are). Thus sufficient repeatability is based on having ICC above a certain threshold rather than demonstrating that the differences are small enough to be biologically unimportant. For example, one study, [1] reported that heel height had a low intraclass correlation coefficient (0.5), which could be improved by training (to 0.75), but such an analysis cannot identify whether the results from one rater could be used interchangeably with those of another (the same issue applies in regard to the concordance correlation coefficient calculated for this study). This is particularly an issue where two different methods are used and their agreement needs to be compared to that of two separate measurements using the standard method, as was the case for this study. This is because ICC calculation is not suitable for method comparison studies as the methods are not chosen at random and could have different measurement-associated errors [22]. In such cases, assessment of mean difference and the variation of that mean (limits-of-agreement) is the optimal method for allowing the differences between the two methods to be compared to the expected measurement error for the standard technique.

There is clearly a need for more analysis of the variation between measurements for all measures of hoof conformation (both between measures by the same individual and between individuals) as well as further research comparing different methods of measurement. This may be particularly important for toe angle which is probably the most difficult to measure of the conformation measures used in this study and also the one where digital measurement showed the best agreement.

## 5. Conclusions

In this analysis digital and manual measures of claw conformation were not equivalent or, for 4/5 measures, interchangeable. Thus, categorizations based on actual measures, such as whether toes are short or long, should not be transferred from manual to digital measures and vice-versa; and comparisons of conformation between studies should be not be made if one study relied on digital measurement and the other on manual measurement

Further research is required to better establish the comparability of all conformation measures so that the agreement between different methods (such as digital *vs.* manual measurement) can be better compared to the expected variation from repeated measures of the same subject using the same method. Such analyses need to take account of differences between individual claws.
